# Recurrent lumbar-origin osteoblastoma treated with multiple surgery and carbon ion radiotherapy: a case report

**DOI:** 10.1186/s12891-020-03349-4

**Published:** 2020-05-22

**Authors:** Akira Honda, Yoichi Iizuka, Reiko Imai, Masahiro Nishinome, Junko Hirato, Hiromi Koshi, Tokue Mieda, Hiroyuki Sonoda, Sho Ishiwata, Yohei Kakuta, Tsuyoshi Tajika, Hirotaka Chikuda

**Affiliations:** 1 Department of Orthopaedic Surgery, Gunma University Graduate School of Medicine, 3-39-22, Showa, Maebashi, Gunma 371-8511 Japan; 2 Hospital of the National Institute of Radiological Sciences, Quantum and Radiological Science and Technology, 4-9-1, Anagawa, Inage, Chiba, 263-8555 Japan; 3 Department of Orthopaedic Surgery, Gunma Rehabilitation Hospital, 2136, Kamisawatari, Nakanojo, Gunma 377-0541 Japan; 4 Clinical Department of Pathology, Gunma University Hospital, 3-39-22, Showa, Maebashi, Gunma 371-8511 Japan

**Keywords:** Recurrent osteoblastoma, Multiple surgery, Carbon ion radiotherapy

## Abstract

**Background:**

Although osteoblastoma is an uncommon benign bone tumor, it sometimes behaves in a locally aggressive fashion. We herein report a case of recurrent lumbar spine osteoblastoma that was treated by repeated surgery and carbon ion radiotherapy.

**Case presentation:**

A 13-year-old Japanese girl presented with left side lumbar pain. Computed tomography and magnetic resonance imaging of the lumbar spine demonstrated a tumorous lesion in the left side pedicle of L4. Although gross total resection of the mass, including the nidus, was performed in the initial surgery, recurrence was observed repeatedly in the short term and the pathological diagnosis of all of the resected tumors was conventional osteoblastoma. We finally performed carbon ion radiotherapy after the patient’s 3rd palliative operation, and achieved a good outcome. No further recurrence has been observed in 10 years of follow-up.

**Conclusion:**

We performed carbon ion radiotherapy for a case of recurrent spinal osteoblastoma and achieved a good outcome without recurrence at 10 years after carbon ion radiotherapy treatment. To the best of our knowledge, this is the first case of osteoblastoma that was treated with carbon ion radiotherapy after multiple surgeries.

## Background

Osteoblastoma (OB) is an uncommon benign bone tumor that accounts for 1% of all primary bone tumors, and approximately 30–40% of all OBs occur on the spine [1, 2]. It is well known that OBs tend to behave more aggressively than other benign bone tumors [3]. Although total resection of the tumor is now the main treatment for cases of symptomatic spinal OB, it is often difficult to complete total resection because of adhesion between the tumor and the surrounding tissues, such as nerve roots or the vertebral artery [1].

Adjuvant chemotherapy and radiotherapy can be considered if OB exhibits an aggressive nature, such as recurrence or malignant change. Furthermore, it has been reported that carbon ion radiotherapy (CIRT) has a cell-killing effect that is 2–3 times greater than X-ray radiotherapy, and also has curative potential in cases involving X-ray-resistant tumors [4, 5]. However, there have been no reports on the use of adjuvant CIRT in the treatment of recurrent lumbar spine OB. We herein report a case of recurrent lumbar spine OB that was treated by repeated surgery and CIRT.

## Case presentation

The patient was a 13-year-old Japanese girl who presented with left side lumbar pain that had persisted for 7 months. She also complained of pain and torpor of the left lower limb for 2 weeks prior to admission for the initial surgery. A neurological examination revealed that the muscle strength of the left side iliopsoas, quadriceps femoris, and tibial anterior muscles was 4/5, 4/5, and 5/5, respectively.

**Fig. 1:**
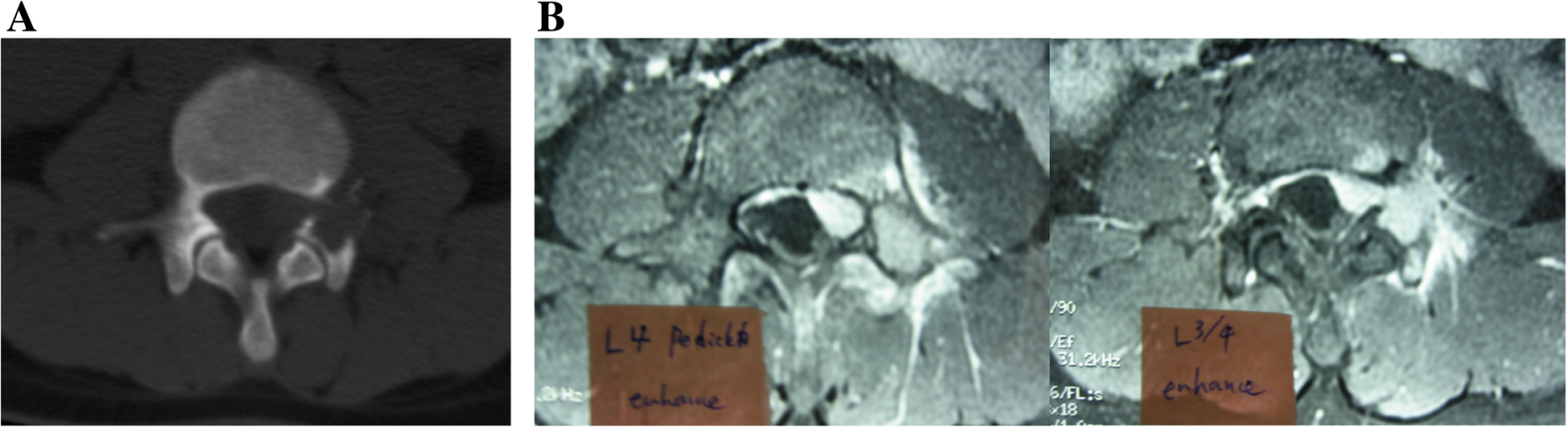
**a** Axial CT of L4 (performed at a previous hospital) showed an osteolytic lesion around the left pedicle. The cortex was seen to be broken by the tumor. **b** T1 axial MRI of the L4 and L3/4 level with Gd enhancement. The left pedicle and its surrounding tissues show enhancement, while the tumor extends to the epidural space

An X-ray examination revealed no remarkable space occupying lesions. Computed tomography (CT) showed an osteolytic lesion on the left side of the L4 pedicle (Fig. 1a). Magnetic resonance imaging (MRI) showed a tumorous lesion extending from the L4 pedicle to the epidural space (Fig. 1b). CT-guided biopsy was performed. Histologically, the tumor showed typical features of osteoblastoma, which was composed of woven bone trabeculae rimmed with a single layer of osteoblasts. There was no marked nuclear pleomorphism or mitotic activity.

**Fig. 2:**
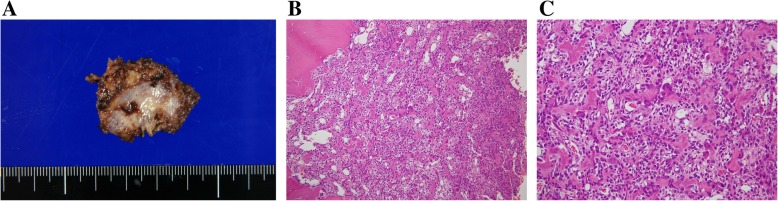
**a** A resected specimen (3 cm × 3.5 cm) at the initial surgery. *En-bloc* excision was performed. **b** A low-power photomicrograph showing irregular trabeculae and prominent vessels. **c** A high-power photomicrograph showing irregular woven bone with osteoblastic rimming. No remarkable large, plump osteoblasts or atypical nuclei were observed, and many small blood vessels between the trabecular bones are seen with no atypia. The pathological findings did not support a diagnosis of osteosarcoma nor epithelioid osteoblastoma

We performed left side facetectomy and gross total resection of the tumor (Fig. 2a), and pathological diagnosis was OB without malignant features. The tumor was composed of irregular woven bone trabeculae. These woven bone trabeculae were lined with a single layer of osteoblasts with no conspicuous permeation to the surrounding bone. Osteoclast-type, multinucleated giant cells without atypia were present. Almost all of the tumor cells were p53-negative (Fig. 2b, c).

**Fig. 3:**
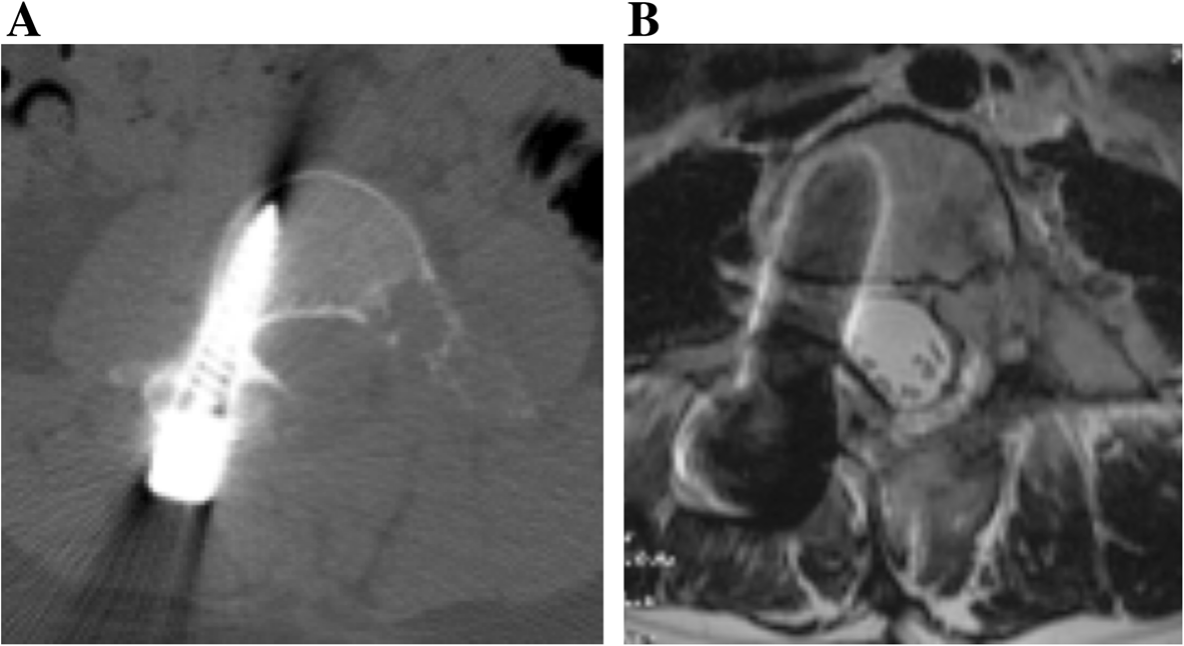
**a** Axial CT of L4 obtained at approximately 4 months after the initial surgery, at the recurrence of lumbago. Recurrence was suspected. **b** The same level on T2 axial MRI

Although her symptoms showed transient improvement, she complained of left side lumbar pain again at 4 months after surgery. CT and MRI showed local recurrence of the tumor (Fig. 3). Based on the radiological findings, we performed a second surgery. However, it was difficult to perform gross total resection of the tumor because of adhesion between the tumor and surrounding tissues. Histologically, the tumor showed the same features as the previous one: OB without malignant change.

**Fig. 4:**
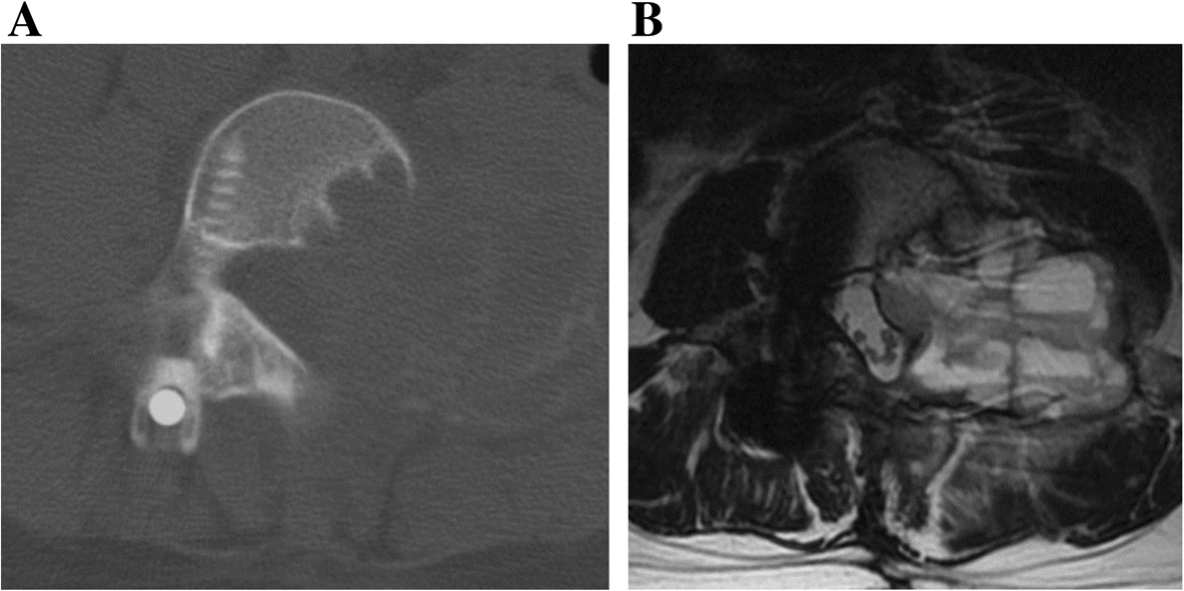
**a** Axial CT of L4 obtained 3 months after the 2^nd^operation. The region extended from the epidural space to the paravertebral area. **b** The same level on T2 axial MRI showed a fluid-fluid level containing lesion. Re-recurrence was suspected

**Fig. 5:**
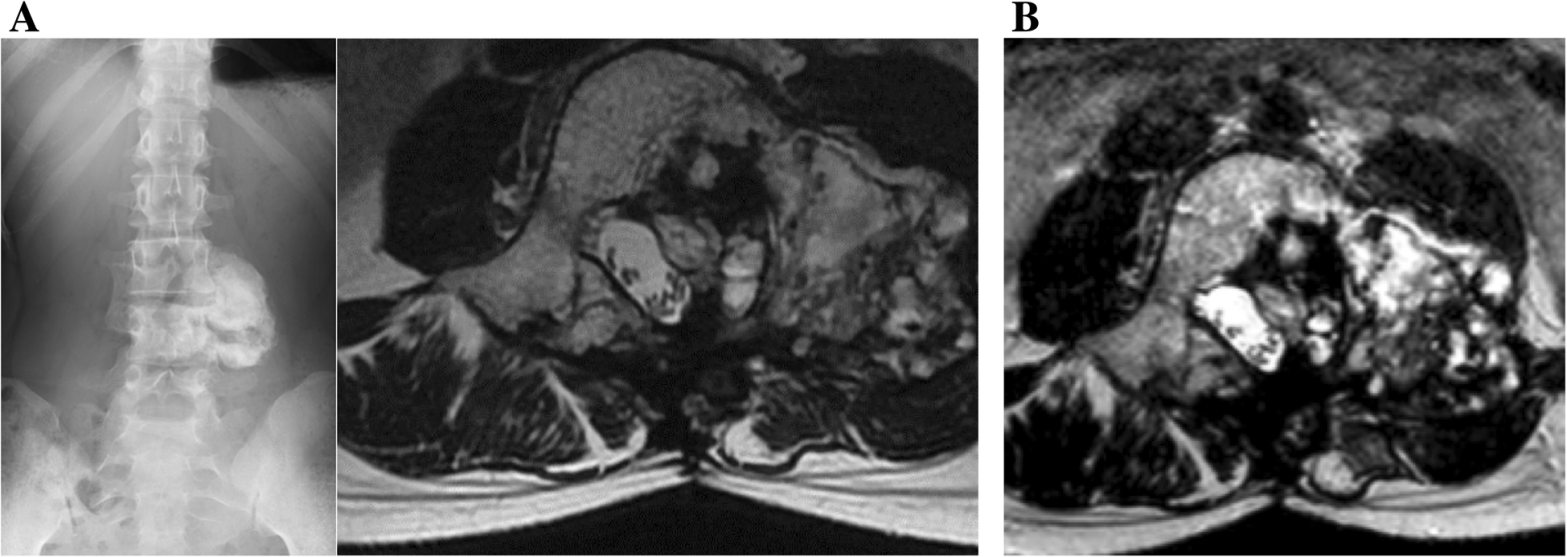
**a** X-ray and T2 axial MRI at 2 years after carbon ion radiotherapy. **b** The same level of T2 axial MRI 10 years later. No significant recurrence was observed

At 3 months after the second surgery, the lumbar pain and pain of the left lower limb recurred. CT and MRI showed a recurrent tumor extending from the epidural space to the paravertebral area (Fig. 4a, b). We performed palliative resection of the tumor due to the worsening of lumbar pain and grade 4/5 muscle weakness. The pathological diagnosis was OB with no malignant change. Hematoma and mild fibrosis were also observed, suggesting ABC-like changes. There were no obvious features of telangiectatic osteosarcoma. CIRT (64 Gy [RBE] in 16 fractions) was performed over 4 weeks as an additional treatment for the residual lesion after the third surgery, because the tumor was locally aggressive. At 10 years after CIRT following multiple surgeries, MRI and X-ray showed neither tumor recurrence nor postoperative spinal deformity and the patient was ambulatory with slight muscle weakness of the left lower limb (Fig. 5a, b, c).

## Discussion and conclusions

In the 2013 World Health Organization Classification of Tumor of Soft tissue and Bone, OB was classified as an intermediate (locally aggressive) osteogenic tumor [6]. At first, OB was reported as a benign osteogenic tumor with a similar histology to osteoid osteoma by Jaffe and Lichtenstein [1, 7]. In 1984, Dorfman and Weiss described some cases of osteoblastoma with infiltrative features and a high rate of recurrence; they referred to those tumors as aggressive OB [8]. Histologically, aggressive OB shows large, plump osteoblasts with prominent nucleoli, called epithelioid osteoblasts. There is no evidence that aggressive OB has a worse prognosis than the standard type of OB and it is debatable whether aggressive OB represents a distinct entity because of its local recurrence. Malignant change of OB was thought to be almost non-existent. These findings indicate that the tumors in cases in which malignant change of OB was reported might have actually been osteosarcoma from the beginning [9–11]. Although surgical treatment results in good outcomes without local recurrence in most cases of OB, the local recurrence rate of OBs after surgery has been reported to be approximately 15–20% [2]. Furthermore, tumors located near important tissues, including the central neural axis, nerve root and vertebral artery have a poor outcome after surgery, probably due to the difficulty associated with completing total resection [1, 2, 6, 12].

Recurrent tumors, especially those located near the spine, are very difficult to treat surgically [13, 14]. Thus, if the tumor has an aggressive nature, adjuvant chemotherapy and radiotherapy are considered after surgery, despite a lack of evidence to support this strategy [15, 16]. Yin et al. reported that the rate of recurrence in patients with aggressive OB was still as high as 60% after radiation therapy following total tumor resection [17]. Although there have been no reports on the effectiveness of CIRT for recurrent OB, CIRT shows great potential as a cure for X-ray-resistant tumors due to its ability to cause complex DNA double-strand breaks [4], and the effectiveness for sarcoma has been described elsewhere [18–20]. In the present case, CIRT was performed instead of conventional X-ray radiotherapy because recurrence developed after an extremely short interval, despite being histologically typical OB, and because it was considered to be a locally aggressive tumor that with aggressive clinical behavior that could not to be controlled by conventional radiation therapy.

**Fig. 6:**
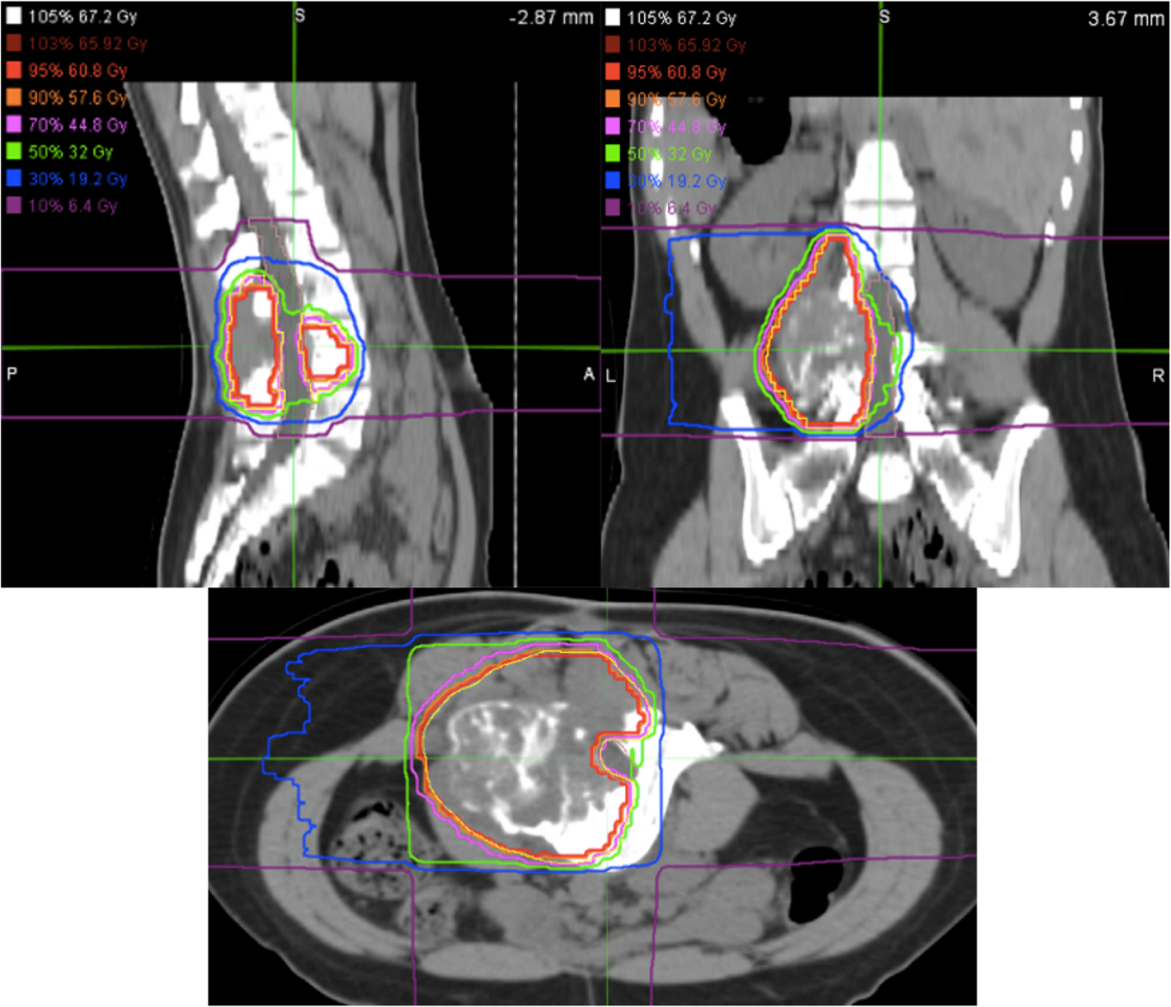
Dose distribution images of CIRT in the case. A total dose of 64.0 Gy (RBE) was applied to the tumor in 16 fractions over 4 weeks (the red line indicates a 90% isodose of the prescribed dose)

The precise irradiation margin remains controversial, especially in the case of spinal tumors. According to previous studies, a margin of 5-mm is considered to provide safety from positioning errors and potential microscopic invasion, irrespective of the histology, and the potential area of spread [18, 19]. However, when the tumor is located close to critical organs, such as the spinal cord, the margin must be reduced accordingly [18]. In the present case, the tumor was close to the cauda equina. Since the dose constraint of the cauda equina is much higher than that of the spinal cord and CIRT can leave the unirradiated area of the cauda equina unirradiated, the coverage of the clinical target volume was prioritized over the dose of the cauda equina (Fig. 6).

The subtypes and transformation of OB are still controversial [12, 21]. In the present case, recurrence was observed repeatedly in a short period after surgery, despite the gross total resection of the tumor in the initial surgery. However, all resected specimens showed the standard type of osteoblastoma, with no signs of malignancy (e.g., no marked nuclear pleomorphism or mitosis) and almost all tumor cells were p53-negative, which suggests osteosarcoma. Furthermore, there was no conspicuous proliferation of epithelioid osteoblasts suggestive of “epithelioid” OB with aggressive nature. Consequently, the addition of CIRT to treat the locally aggressive tumor after multiple surgeries achieved a good outcome.

In conclusion, the possibility of clinically aggressive behavior of OB should be taken into account, even when the histology is not consistent with malignancy or “epithelioid” OB. CIRT can be a treatment option for spinal OBs with a clinically aggressive nature, such as OB in cases with repeated recurrences soon after surgery.

## Data Availability

The datasets used and/or analyzed during the current study available from the corresponding author on reasonable request.

## Abbreviations

OB: Osteoblastoma
CIRT: Carbon ion radiotherapy
CT: Computed tomography
MRI: Magnetic resonance imaging
Gy: Gray
RBE: Relative biological effectiveness
